# Intensive Periodontal Treatment Does Not Affect the Lipid Profile and Endothelial Function of Patients with Type 2 Diabetes: A Randomized Clinical Trial

**DOI:** 10.3390/biomedicines10102524

**Published:** 2022-10-09

**Authors:** Biagio Rapone, Elisabetta Ferrara, Erda Qorri, Mir Faeq Ali Quadri, Gianna Dipalma, Antonio Mancini, Massimo Del Fabbro, Antonio Scarano, Gianluca Tartaglia, Francesco Inchingolo

**Affiliations:** 1 Interdisciplinary Department of Medicine, “Aldo Moro” University of Bari, 70121 Bari, Italy; 2 Complex Operative Unit of Odontostomatology, Hospital S.S. Annunziata, 66100 Chieti, Italy; 3 Dean Faculty of Medical Sciences, Albanian University, Bulevardi Zogu I, 1000 Tirana, Albania; 4 Department of Preventive Dental Sciences, College of Dentistry, Jazan University, Jazan 45142, Saudi Arabia; 5 UOC Maxillo-Facial Surgery and Dentistry, Fondazione IRCCS Ca Granda, Ospedale Maggiore Policlinico, 20122 Milan, Italy; 6 Department of Biomedical, Surgical and Dental Sciences, University of Milan, 20122 Milan, Italy; 7 Department of Oral Science, Nano and Biotechnology and CeSi-Met University of Chieti-Pescara, 66100 Chieti, Italy

**Keywords:** endothelial dysfunction, periodontitis, periodontal disease, periodontal therapy, oxidative stress, biomarkers

## Abstract

**Background:** Local eradication of periodontal infection could potentially have a much broader impact on the diabetic condition by also contributing to the modification of the lipid profile, which is directly compromised in the alteration of endothelium-dependent vasodilation. The aim of this trial was to assess the benefits of intensive periodontal treatment (IPT) on the lipid profile and endothelial function of diabetic patients. **Methods:** This was a 6-month, randomized controlled trial involving diabetic patients with generalized periodontitis. The study group comprised 290 individuals who were randomly assigned to receive Intensive Periodontal Treatment (IPT, Intervention Group) or conventional adult prophylaxis (Control Periodontal Treatment, CPT, Control Group). Outcomes encompassed lipid profile involving serum total cholesterol, serum triglyceride, low-density lipoprotein cholesterol, high-density lipo-protein cholesterol, and flow-mediated vasodilation (FMD) as an index of endothelium-dependent vasodilation (primary outcomes); periodontal indices and high-sensitive C-reactive protein were evaluated at baseline, 3 and 6 months after periodontal treatment. **Results:** An increase in endothelium-dependent flow-mediated dilatation (FMD) was observed in the Intensive Periodontal Treatment group in comparison with Control (*p* < 0.001), but results are not statistically different. There were no differences in lipid profile in individuals of both groups. **Conclusions:** An intensive periodontal treatment might improve endothelial function, suggesting a direct beneficial effect on the vasculature, possibly mediated by systemic inflammatory reduction. However, no statistically significant differences between groups were observed, and no benefits were proved on lipid profile.

## 1. Introduction

Periodontal disease (PD) and Diabetes Mellitus (DM) are non-communicable chronic diseases related by a common immuno-regulatory dysfunction. Several studies have provided compelling evidence on the bidirectional relationship between periodontitis and diabetes [1,2,3,4]. This is further established in the consensus report and guidelines of the International Diabetes Federation and the European Federation of Periodontology of 2018 [5]. Periodontitis is an infectious–inflammatory disease triggered by bacterial plaque and concomitant predisposing factors, which appears as the progressive alteration of the architecture and functionality of the periodontal tissues [6]. These pathological changes originate from the activation of inflammatory pathways that promote an adverse positive feedback loop that upregulates the release of pro-inflammatory subset, which generates the chronicization of the phlogistic condition [7,8,9]. This peripheral process triggers systemic inflammatory signaling pathways, contributing to insulin resistance. The risk of periodontitis is three-fold higher in diabetic individuals than in healthy individuals, and subjects with periodontitis have an increased risk of dysglycaemia and insulin resistance [10,11,12]. Results from multiple clinical trials confirm that glycated hemoglobin levels are significantly higher in diabetic patients with periodontitis than in healthy control subjects and that periodontal healing is associated with the reduction in blood glycated hemoglobin concentrations. Concomitantly, the resolution of periodontal infection in individuals with diabetes and periodontitis has shown a significant reduction in high-sensitive C-reactive protein (hs-CRP) levels [10,13,14,15]. Furthermore, the potential impact of periodontitis on lipid profile and any associated consequences on the endothelial function is attracting interest [16]. It has been shown that the co-existence of periodontitis not only affects the insulin resistance in people with diabetes but also might interfere with endothelial function, also through the alteration of lipid profile [16,17]. Periodontal treatment has been suggested to have a potential role in decreasing endothelial dysfunction by modulating the immune response and glycemic level in diabetic patients, as well as through the improvement of lipid profile [17,18].

There are some randomized clinical trials (RCTs) that have found an improvement in lipid profile after periodontal treatment [16,18]. Lipid disorders are associated with the endothelium damage and impairment of HDL function [16]. Therefore, periodontal treatment could potentially reduce the level of elevated lipids levels and subsequently reduce the endothelial dysfunction. Since there are few studies assessing the lipid profile and concomitant endothelial dysfunction in diabetes patients undergoing periodontal treatment, the purpose of this study was to assess the changes in lipid profile in diabetic patients after intensive periodontal treatment. 

## 2. Materials and Methods

### 2.1. Study Design

A prospective, single-blinded, randomized controlled trial, comparing conventional periodontal treatment versus intensive periodontal treatment for patients with type 2 Diabetes, with a follow up of six months post-treatment, was conducted between September 2019 and October 2020. Prior to starting the study, ethical approval was obtained from the local Institutional Review Board of Albanian University (Reference Number 2018/114). The trial is registered with the ISRCTN registry, number ISRCTN69139365. Participants recruited provided written consent before participation. This study was executed in accordance with the guidelines of the Declaration of Helsinki. Written informed consent was obtained from all subjects participating in the trial prior to randomization. 

### 2.2. Sample Size Calculation

A sample size calculation was conducted to estimate the number of participants required for the final study analysis. The sample size was established considering a number of groups equal to two, an effect size of 0.30 for flow-mediated dilatation (FMD), a two-sided significance level of 0.05, and a power of 80%. It was estimated that ≈120 patients per group would be needed. Considering a 20% drop-out rate, 290 participants were considered adequate. 

### 2.3. Screening, Enrolment, and Randomization

Patients diagnosed with type 2 Diabetes receiving diabetes care at the outpatient clinic were screened for eligibility. Participants were included if they met the following criteria: (1) older than 18 years, (2) having generalized periodontitis (presence of at least 16 teeth; (3) a minimum of 40% of sites with a clinical attachment level (CAL) ≥2 mm and probing depth (PD) ≥4 mm; (4) presence of at least ≥2 mm of crestal alveolar bone loss established with digital periapical radiographs; (5) presence of ≥40% sites with bleeding on probing (BOP); and (6) willingness to give informed consent to participate in the study. Patients who received periodontal treatment within 12 months prior to the start of the study, as well as patients who received systemic antibiotics within the last 6 months, having uncontrolled hypertension, pregnant, history of heart disease or stroke, breast-feeding women, and patients unable to make informed consent were excluded. After baseline assessments, patients were randomly allocated to either a Control or Intervention group using computer-generated random numbers. The study was set up as a single-blind trial with the investigators assessing outcomes blinded to treatment allocation throughout the trial. Due to the nature of the interventions, study participants and periodontists were not masked for the treatment protocol. Randomization resulted in an Intervention group and a Control group followed-up by specialists in periodontology. All patients were subject to the ordinary routine visits to the diabetic out-patient clinic and to periodontal examination at baseline, 3, and 6 months post-randomization. Thus, 145 patients were enrolled in the Intervention and Control groups and were followed-up at 6 months post-randomization.

### 2.4. Outcomes

The primary outcomes were the change in lipid profile and the change in flow-mediated dilation of the brachial artery (FMD), as a measure of endothelial dysfunction. The secondary outcomes were the differences in hs-C-reactive protein (hs-CRP), glycated hemoglobin levels, and periodontal indices.

### 2.5. Assessment Criteria

#### 2.5.1. Biomarkers 

Glycated hemoglobin (HbA1c), triglycerides (TG), total cholesterol (TC), low-density lipoprotein cholesterol (LDL-C), and high-density lipoprotein cholesterol (HDL-C) were tested at local primary care center using standard methods at baseline, 3, and 6 months. Biomarkers were determined by enzyme-linked immunosorbent assay (ELISA) technique using a test system from R&D (Minneapolis, MN, USA).

#### 2.5.2. Endothelial Function

Flow-mediated dilation was assessed according to current guidelines [19]. Endothelial function was assessed non-invasively using the flow-mediated dilation (FMD) technique. The dilatation of the brachial artery is affected by drugs, temperature, stress, food, and sympathetic system tone. It is suggested to standardize the examination conditions, so the subjects avoided food for 8–12 h before the examination in a room with a constant temperature between 22 °C and 24 °C. The measurement was performed on the same arm using a high-resolution US instrument. Patients were positioned with their right arm extended at an angle of ~80° from the torso and immobilized with foam supports. After the acquisition of the baseline image, the brachial artery was closed by cuff inflation with pressure above the systolic value. It was inflated at the same supra-systolic pressure to occlude arterial inflow for a standardized period of time. After cuff deflation, the image of the brachial artery was recorded continuously by the US from 30 s before until 3 min. The baseline value is the average of the measurements during the first minute after cuff deflation. The ratio between the maximum diameter after the cuff deflation and the basal diameter of the artery is the reactive increase in flow. FMD was calculated as the percentage increase in the diameter of the artery after application of the pressure stimulation. The FMD of the brachial artery was assessed by means of ultrasound imaging (Acuson XP 128/10, Siemens, USA/Canada) with the use of a 7-MHz linear probe and automated vessel diameter measurements (Brachial Tools, version 3.2.6, Medical Imaging Applications, USA/Canada) as previously described [19].

#### 2.5.3. Periodontitis

After completing the endothelial function evaluation, subjects underwent their baseline basic periodontal examination (BPE), a mandatory procedure in diagnosing periodontitis, to collect the following information about the periodontal status: probing pocket depth (PPD), which is defined as the distance between the gingival margin and the base of the sulcus/pocket; clinical attachment level (CAL), which is determined by calculating the distance between the base of the pocket and the cemento-enamel junction; gingival index (GI), which refers to the cardinal signs of inflammation; and plaque index (PI), which refers to the amount of dental/bacterial plaque [20,21]. Examinations were executed by employing a graduated periodontal probe (Michigan O probe with Williams’s markings) along the axis of the tooth into the gingival sulcus, at six sites per tooth, applying a slight pressure of 0.25 N [22]. PPD and CAL are the pathognomonic signs of periodontal disease, whereas gingival index (GI) and plaque index (PI) were recorded to assess the gingival inflammatory status. The degree of periodontitis was assessed according to the Consensus Report of World Workshop on the Classification of Periodontal and Peri-Implant Diseases and Conditions [23]. Specialists in periodontology were trained to assess the degree of periodontitis. The intra- and inter-rater reliability was assessed using the intra-class correlation coefficient (95% confidence interval (95% CI) 0.981–0.990); *p*  <  0.0001. 

### 2.6. Periodontal Treatment

Before treatment, patients of both groups received oral hygiene instructions. All study participants underwent Intensive Periodontal Treatment (IPT) or Control Periodontal Treatment (CPT) within 1 month from the baseline visit. The protocol of IPT consisted of a single session of one-stage full-mouth disinfection (OSFM), which implicated supra- and sub-gingival mechanical debridement scaling and manual root surface and calculus removal of all pockets (root planing), under local analgesia, within 24 h and in association with chlorhexidine application to all oropharyngeal niches (chairside and at home for 2 months after treatment) [20]. Additionally, periodontal surgery was executed for periodontal pockets >5 mm or residual periodontal pockets. The CPT included supra-gingival mechanical debridement. The root planing was delayed at 6 months after completion of the trial. At 3 months, supra- and sub-gingival mechanical debridement scaling was executed for both the groups.

### 2.7. Statistical Analysis

**Figure 1:**
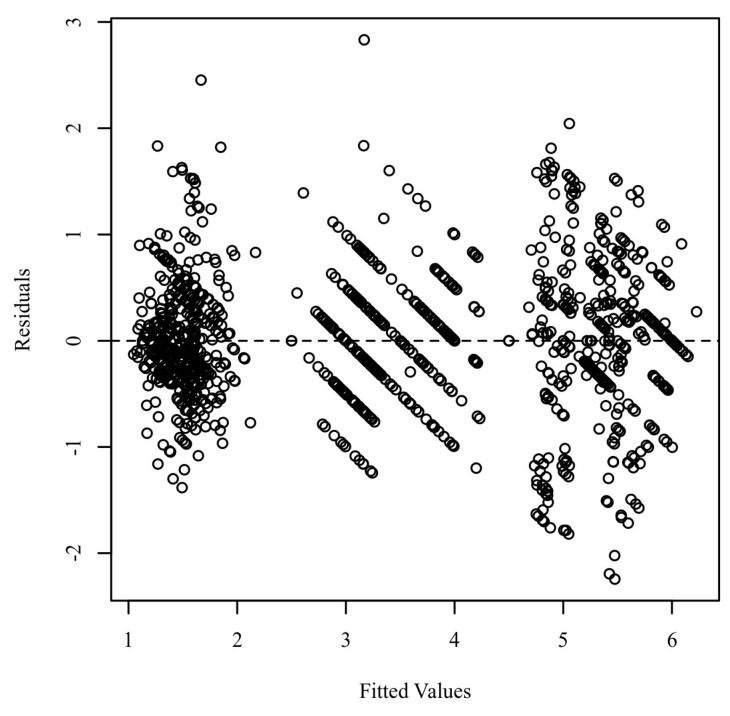
Residuals scatterplot testing homoscedasticity.

For normally distributed data, continuous variables were expressed as mean ± standard deviation or median and interquartile range (IQR), whereas categorical variables were given as frequencies and percentages. The normality of continuous parameter distribution was checked using Skewness and Kurtosis. The comparison of change in primary and secondary outcomes between the two groups at 3 and 6 months was conducted using the two-tailed Wilcoxon signed rank test. A mixed-model multivariate analysis of covariance (MANCOVA) with two within-subjects factors and one between-subjects factor was conducted to compare and to examine whether significant differences existed among the time points for the variables for both groups. Cohen’s standard was used to evaluate the strength of the relationships. Post hoc univariate F tests were performed to analyze separately each component of the dependent variable. Homoscedasticity was evaluated by plotting the residuals against the predicted values. Figure 1 presents a scatterplot of predicted values and model residuals.

The assumption of sphericity was assessed using Mauchly’s test. To identify influential points in the residuals, Mahalanobis distances were calculated and compared with an χ^2^ distribution. The assumption for homogeneity of regression slopes was assessed by rerunning the mixed model MANCOVA, but this time including interaction terms between each independent variable and covariate. The covariate analysis was conducted at baseline. An ANOVA was conducted for each pair of numeric covariates and independent variables to assess independence. A multinomial regression model was conducted and compared with the null model for each pair of categorical covariates and independent variables to assess independence. The Bonferroni test was used for post hoc analysis. For all analyses, 95% confidence intervals were presented for each measurement time-point. The level for statistical significance was accepted to be *p* < 0.05. Data analysis was performed by IBM SPSS Statistics 21.0 (IBM Corp. Released 2012. IBM SPSS Statistics for Windows, Version 21.0. Armonk, NY, USA: IBM Corp.).

## 3. Results

**Figure 2:**
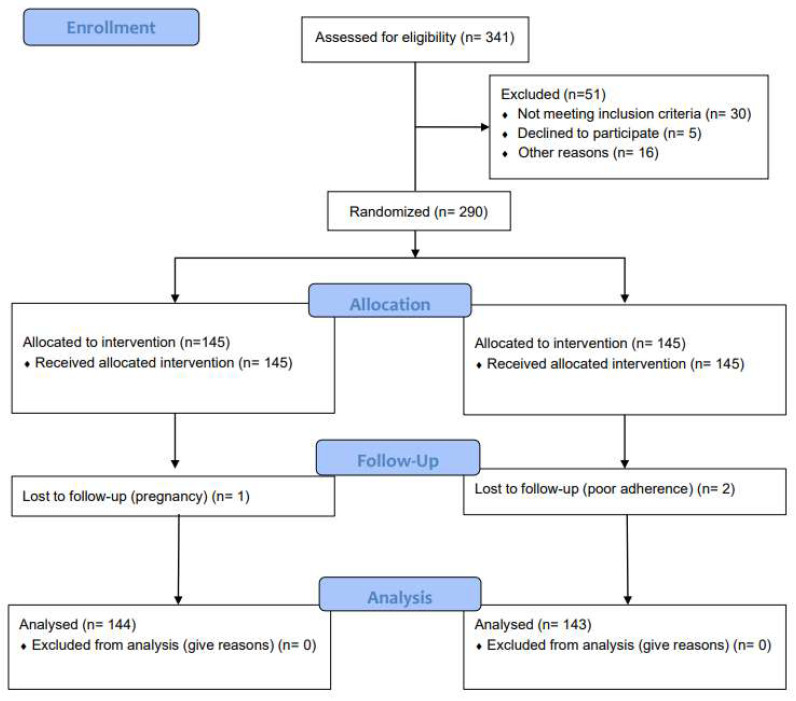
The study flow-chart.

Trial recruitment started in April 2020 and follow-up was concluded in October 2021. During this period, 341 patients were screened for eligibility. A total of 290 subjects were enrolled. In the Intervention group, out of 145 participants, 1 dropped out due to pregnancy. In the Control group, two patients dropped out during the follow-up, manifesting poor adherence to treatment. In total, 144 and 143 subjects in the Intervention and Control groups, respectively, were included for the final data analysis. There were no documented changes in the physical activity and smoking status during the study period, as required. Figure 2 shows the flow-chart of trial participants. 

Baseline characteristics of the participants enrolled at the start of the study are summarized in Table 1. 

As shown in Table 2 and Table 3, the differences between the median of FMD for the Intervention Group and Control Group were not statistically different. 

**Figure 3:**
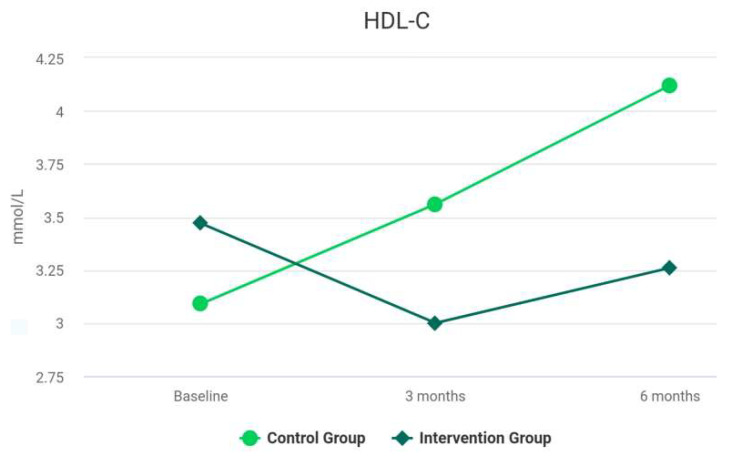
Results of HDL-C at baseline and during follow-up.

**Figure 4:**
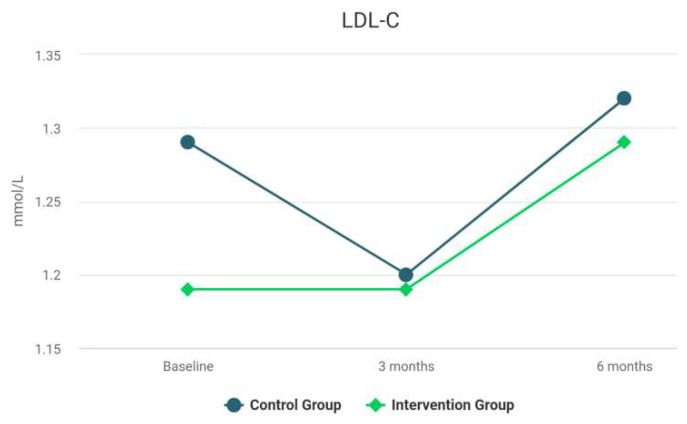
Results of LDL-C at baseline and during follow-up.

**Figure 5:**
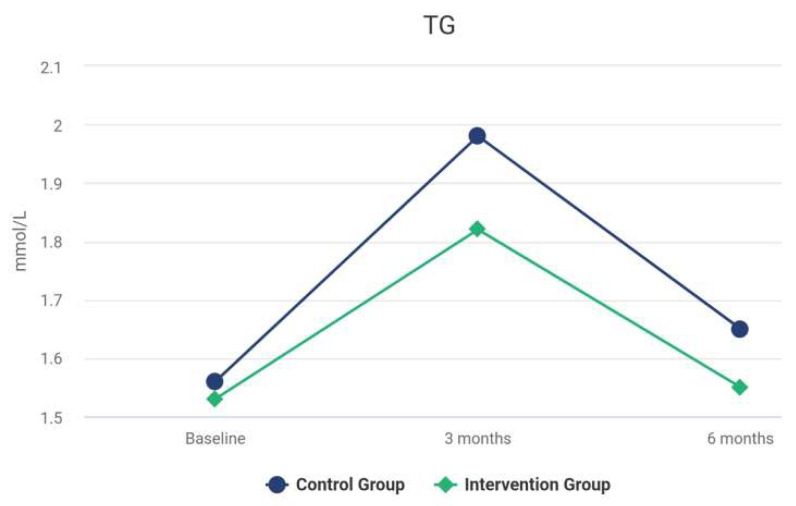
Results of TG at baseline and during follow-up.

**Figure 6:**
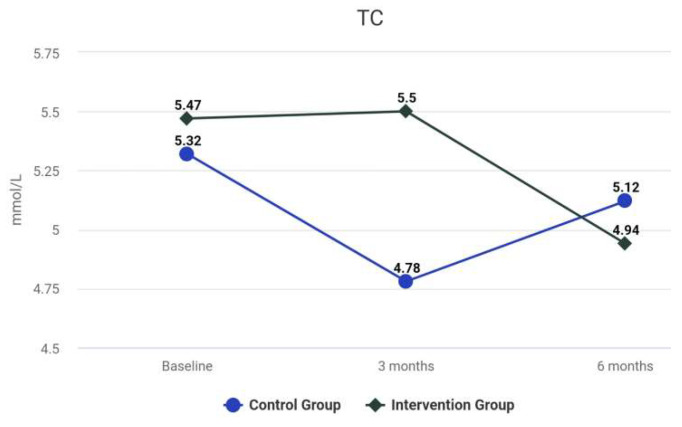
Results of TC at baseline and during follow-up.

**Figure 7:**
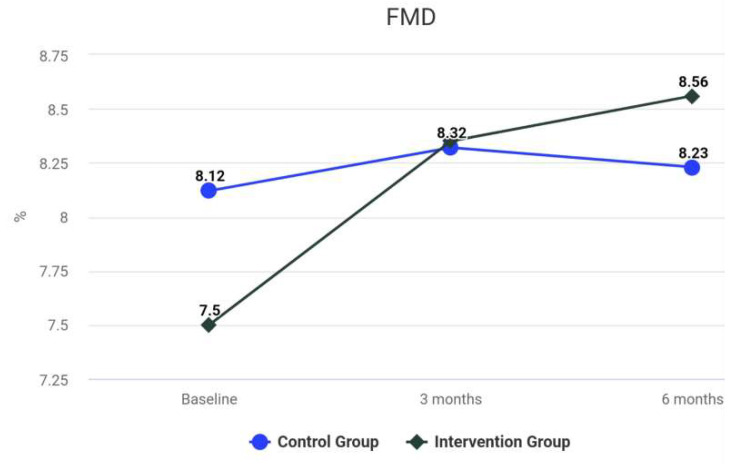
Results of TC at baseline and during follow-up.

**Figure 8:**
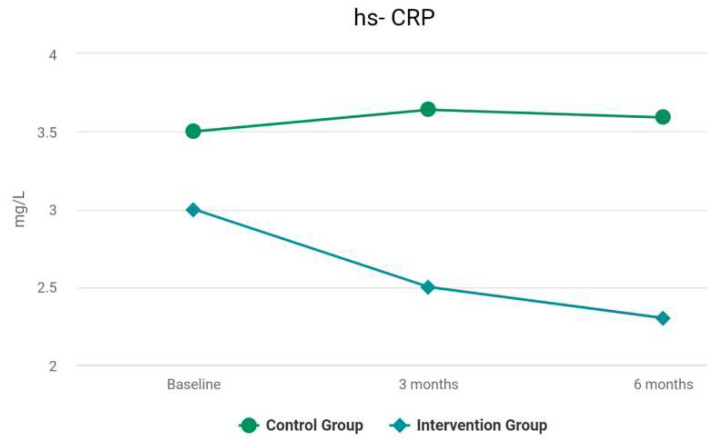
Results of hs-CRP at baseline and during follow-up.

The difference between the groups at 6 months was not statistically significant. The results for the Intervention group reveal a significant improvement in hs-CRP over time. There was a statistically significant difference between the hs-CRP levels at baseline and the values at 3 months, *z* = −3.71, *p* < 0.001. The difference between the results at 3 and 6 months was not statistically significant (*z* = −0.39, *p* = 0.694). For the Control group, the difference between the rates was not statistically significant before and after the treatment (*z* = −0.29, *p* = 0.592). The differences between the groups at 3 and 6 months were statistically significant, *z* = −1.59, *p* = 0.118. At 3 months after the treatment, the differences between the groups for LDL-C were not significant, *V* = 463.00, *z* = −0.12, *p* = 0.904. The median of HDL-C of the *Control* Group at 3 months was significantly larger than the median of HDL-C of the *Intervention* Group (*V* = 690.00, *z* = −2.62, *p* = 0.009). As shown in Table 2, at 3 months, no statistically significant difference of serum levels of HbA1c between the groups was observed (*z* = −1.57, *p* = 0.116). As shown in Table 2, analyses revealed that the TC levels, at 3 and 6 months, were not statistically different among the groups, *p* = 0.923, and *p* = 0.867, respectively. The levels of TG were significantly reduced after intensive periodontal treatment in comparison with the Control group at 3 and 6 months, *p* < 0.001. Table 4 presents the MANCOVA results. Differences between the focal indices are shown in Figure 3, Figure 4, Figure 5, Figure 6, Figure 7 and Figure 8.

Between-Subjects: The main effect for IG-CG was not significant *F*(1, 78) = 0.87, *p* = 0.353, indicating the levels of IG-CG were all similar for CAL, PPD, TC, TG, and LDL-C after controlling for duration of diabetes, age, smoking, and physical activity. There were significant differences in HDL-C, HbA1c, hs-CRP, and FMD, with *F*(1, 78) = 57.89, *p* < 0.001, between the levels of IG-CG after controlling for the covariates. The covariate duration of diabetes was not significantly related to CAL, PPD, TC, TG, and LDL-C, *F*(1, 78) = 1.25, *p* = 0.267. The covariate age was not significantly related to CAL, PPD, TC, TG, and LDL-C, *F*(1, 78) = 0.24, *p* = 0.626, to HDL-C, HbA1c, and hs-CRP, *F*(1, 78) = 3.58, *p* = 0.062. The covariate smoking was not significantly related to CAL, PPD, TC, TG, and LDL-C, *F*(2, 78) = 0.15, *p* = 0.859, to HDL-C, HbA1c, and hs-CRP, *F*(2, 78) = 1.32, *p* = 0.274. The covariate physical activity was not significantly related to CAL, PPD, TC, TG, and LDL-C, *F*(2, 78) = 0.39, *p* = 0.680, to HDL-C, HbA1c, and hs-CRP, *F*(2, 78) = 0.45, *p* = 0.642. 

Within-Subjects: The main effect for Time Factor was significant *F*(2, 156) = 12.73, *p* < 0.001, indicating there were significant differences in CAL, PPD, TC, TG, LDL-C, HDL-C, HbA1c, and hs-CRP across the levels of Time Factor ignoring dependent variables after controlling for covariates. The main effect for dependent variables was significant *F*(4, 312) = 12.99, *p* < 0.001, indicating there were significant differences across the levels of dependent variables, CAL, PPD, TC, TG, LDL-C, HDL-C, HbA1c, and hs-CRP, regardless of Time Factor after controlling for covariates. The interaction effect between Time Factor and dependent variables was significant *F*(8, 624) = 7.67, *p* < 0.001, indicating that the relationships between the levels of dependent variables differed significantly across the levels of Time Factor after controlling for covariates.

Within-Between Interactions: The interaction effect between Time Factor IG-CG was not significant *F*(2, 156) = 2.77, *p* = 0.070, indicating that the relationships between the levels of Time Factor were similar between the levels of IG-CG, ignoring dependent variables after controlling for covariates. The interaction effect between dependent variables and IG-CG was not significant *F*(4, 312) = 1.36, *p* = 0.260, indicating that the relationships between the levels of Dv Factor were similar between the levels of IG-CG, regardless of Time Factor after controlling for covariates. The interaction effect between Time Factor, dependent variables, and IG-CG was significant *F*(8, 624) = 3.49, *p* = 0.006, indicating that the relationships between the combinations of Time Factor and dependent variables differed significantly between the levels of IG-CG after controlling for covariates. 

Within-Covariate Interactions: The interaction effect between Time Factor and duration of diabetes was not significant, *F*(2, 156) = 0.74, *p* = 0.468, indicating that the relationships between the levels of Time Factor were similar for all values of duration of diabetes. The interaction effect between Time Factor and age was not significant, *F*(2, 156) = 2.43, *p* = 0.096. The interaction effect between Time Factor and smoking was not significant, *F*(4, 156) = 0.33, *p* = 0.840. The interaction effect between Time Factor and physical activity was not significant, *F*(4, 156) = 0.22, *p* = 0.917. The interaction effect between Dv Factor and duration of diabetes was not significant, *F*(4, 312) = 0.51, *p* = 0.627, indicating that the relationships between the levels of Dv Factor were similar for all values of duration of diabetes. The interaction effect between dependent variables and age was not significant, *F*(4, 312) = 0.63, *p* = 0.557. The interaction effect between dependent variables and smoking was not significant, *F*(8, 312) = 0.79, *p* = 0.547. Similarly, the interaction effect between dependent variables and physical activity was not significant, *F*(8, 312) = 0.30, *p* = 0.902. No significant interaction effect between Time Factor, Dv Factor, and duration of diabetes was revealed, *F*(8, 624) = 0.52, *p* = 0.739. The interaction effect between Time Factor, dependent variables, and age was not significant, *F*(8, 624) = 1.95, *p* = 0.093. The interaction effect between Time Factor, dependent variables, and smoking was not significant, *F*(16, 624) = 0.78, *p* = 0.632. The interaction effect between Time Factor, dependent variables, and physical activity was not significant, *F*(16, 624) = 0.92, *p* = 0.511.

## 4. Discussion

Our study aimed to examine the impact of periodontal inflammation on lipid profile and endothelial function in patients with diabetes. Endothelial dysfunction is a pathological condition typified mainly by an imbalance of endothelial-dependent relaxing and contracting factors. In diabetes, lipotoxicity has the potential to affect the endothelial cell homeostasis and cause insulin resistance by decreasing endothelial NO synthase (eNOS) gene expression and eNOS catalytic activity. The mechanism linking insulin resistance to endothelial dysfunction is complex and includes the release of excess free fatty acid release. This risk factor, in combination with hypertension, genetic predisposition, hyperglycemia, hyperinsulinemia, oxidative stress, and advanced glycation end products, converges on the artery (center), promoting atherogenesis [24,25]. The correlation between periodontitis and endothelial dysfunction is a debate that is still open, since although the plausibility of this association is known, the causal link has not been identified [1,16,21,22]. Interestingly, diabetes carries with it cardiovascular complication, which in turn has merited attention from scientific research for its potential link with periodontal disease [22,23,24,25,26,27,28]. The potential mechanistic overlap between diabetes, periodontitis, and endothelial dysfunction is through low-grade inflammation and oxidative stress status [5,6,7,8,29,30]. The progressive reduction in hs-CRP in the Intervention Group and the oscillation for the Control group confirmed the effectiveness of periodontal therapy in reducing the inflammatory marker. The results indicate that hs-CRP levels were similar between the groups during the follow-up. 

Periodontal indices were similar between study groups at baseline, while significant differences occurred in the change from baseline to study in the 3- and 6-month median PPD and CAL, respectively, between groups. Seinost et al. [31] found a statistically significant difference in flow-mediated dilation, which was lower in patients with periodontitis compared with the Control subjects at baseline (6.1% F 4.4% vs. 8.5% F 3.4%, *p* = 0.002) and was improved after periodontal treatment, leading to an increase in FMD (9.8% F 5.7%; *p* = 0.003 vs. baseline) along with a reduced significant reduction in C-reactive protein levels. No difference was reported for endothelium-dependent nitric vasodilation. Few studies have explored the potential association between changes in oxidative stress status of diabetic patients with periodontitis and the improvement of endothelial function after intensive periodontal treatment [32,33,34,35,36,37,38,39]. In contrast with our results, previous studies that investigated the impact of intensive periodontal treatment on endothelial dysfunction demonstrated a short-term improvement in FMD after periodontal therapy [10,36,38,39,40,41]. In the study by Masi et al. [42], the impact of periodontal inflammation on diabetes was explored by examining the beneficial effect of periodontal treatment on lipid profile. In this study, patients with diabetes and periodontitis were assigned to intensive periodontal treatment or standard periodontal therapy to evaluate the potential convergence of changes in periodontal status and changes in endothelial function after periodontal therapy. 

One population-based cohort study in Pomerania of 1234 subjects found that periodontal parameters were significantly associated with increased levels of FMD [43]. More recently, Ferlazzo et al. [39] confirmed their findings by adding an examination of patients with simultaneous periodontitis and coronary artery disease. They also noted a significant increase in the concentration of 3-nitrotyrosine (NT), which reinforces the concept of the involvement of periodontal disease in the imbalance of endothelial function. Similarly, HbA1 plasma levels differed significantly during follow up for each group. We examined systemic inflammatory status by quantifying plasma concentrations of hs-CRP, a sensitive marker of odontogenic inflammation in patients with diabetes and cardiovascular disease [11,40,41]. Hs-CRP decreased for each group at each time-point, with statistically significant difference [40]. Our results do not support a statistically significant correlation between markers of endothelial dysfunction and periodontitis. The explanation may lie in two aspects: The first concerns the impact of periodontitis per se. We examined patients with an underlying pathology predisposing them to cardiovascular disease; in this context, the periodontitis phenomenon might be attenuated or concealed due to protracted hyperglycemia [43]. Second, the number of patients observed was relatively low, and the follow-up was too short to appreciate substantial changes. The values of follow-up FMD, low-density lipoprotein (LDL-C), total cholesterol (TC), and triglycerides (TG) between both groups were not statistically significant. A larger sample is needed to define whether intensive periodontal treatment (IPT) might have an impact on lipid profile and endothelial function on patients with diabetes.

## 5. Conclusions

The above observations do not suggest that periodontitis might trigger endothelial dysfunction in diabetic and lipid profile alteration. Future research may help clarify the roles of periodontitis in enhancing the lipid profile and endothelial function of patients with diabetes.

## Figures and Tables

**Table 1:** Patient’s demographic and clinical characteristics at baseline.

Variables	* CG Baseline	** IG Baseline	*p*
Age (mean ± SD)	57.49 ± 8.52	59.12 ± 7.48	0.091
Female, (%)	54	68	0.801
Duration of diabetes (years, mean ± SD)	6.1 ± 5.2	5.5 ± 3.4	0.078
Smoking (%)	53	38	0.087
Physical activity (%)	29	34	0.976
BMI, kg/m^2^ (Mean ± SD)	28.2 ± 1.5	27.9 ± 4.3	0.354
hs-CRP, mg/L (Median, IQR)	3.5 (1)	3 (0.025)	0.654
HbA1c (%) (Median, IQR)	7.75 (2)	8.1 (1.2)	0.643
TC, mmol/L(Median, IQR)	5.32 (1.2)	5.47 (0.13)	0.782
TG, mmol/L (Median, IQR)	1.56 (1)	1.53 (0.06)	0.821
LDL-C, mmol/L (Median, IQR)	1.29 (0.08)	1.19 (0.05)	0.655
HDL-C, mmol/L (Median, IQR)	3.09 (1)	3.47 (0.11)	0.425
FMD (Median, IQR)	8.12 (1)	7.50 (0.25)	0.342
CAL, mm (Median, IQR)	6.2 (0.12)	6 (0.23)	0.455
PPD, mm (Median, IQR)	5.11 (1)	5.42 (0.08)	0.877
PI, % (Median, IQR)	79.56	75.76	0.898
GI, % (Median, IQR)	92.67	87.88	0.849
Blood pressure (mmHg)	124 ± 13	125 ± 19	0.848

Note: * CG, Intervention Group; ** IG, Control Group.

**Table 2:** Two-tailed Wilcoxon signed rank test results at 3 months.

3 Months				
Variable	** CG	* IG	*z*	*p*
HbA1c (%)	8.05 (0.10)	7.8 (0.21)	−1.57	0.116
CAL (mm)	5	3.45 (0.08)	−5.85	<0.001
PPD (mm)	3.4	3.10 (0.23)	−4.87	<0.001
TC (mmol/L)	4.78	5.50 (0.12)	0.11	0.923
TG (mmol/L)	1.98	1.82 (0.07)	−0.12	0.867
LDL-C (mmol/L)	1.20	1.19 (0.07)	−0.12	0.904
HDL-C (mmol/L)	3.56 (1.2)	3.00 (1)	0.28	0.698
FMD	8.32 (0.46)	8.35 (0.12)	0.11	0.677
hs-CRP (mg/L)	3.64 (1.65)	2.5	−1.49	0.112

Note: * IG, *Intervention* Group; ** CG, *Control* Group.

**Table 3:** Two-tailed Wilcoxon signed rank test at 6 months.

6 Months	Median (IQR)			
Variable	* CG	** IG	*z*	*p*
HbA1c (%)	7.30 (0.78)	7.20 (0.21)	−0.34	0.737
CAL (mm)	5	3.00 (0.08)	−1.23	<0.001
PPD (mm)	3.2 (0.8)	2.93 (0.07)	−1.48	0.138
TC (mmol/L)	5.12 (1)	4.94 (1.09)	0.12	0.867
TG (mmol/L)	1.65 (0.9)	1.55 (0.08)	0.14	0.138
LDL-C (mmol/L)	1.32 (1)	1.29 (1.2)	0.16	0.140
HDL-C (mmol/L)	4.12	3.26 (0.10)	−1.21	<0.001
FMD	8.23 (0.46)	8.56	0.12	0.137
hs-CRP (mg/L)	3.59 (1)	2.3	−1.59	<0.001

Note: * CG, Intervention Group; ** IG, Control Group.

**Table 4:** MANCOVA Results.

Source	*df*	*SS*	*MS*	*F*	*p*	η_p_2
Between-Subjects						
IG _CG	1	1.00	1.00	0.87	0.353	0.01
Duration of diabetes	1	1.43	1.43	1.25	0.267	0.02
Age	1	0.27	0.27	0.24	0.626	0.003
Smoking	2	0.35	0.17	0.15	0.859	0.004
Physical activity	2	0.89	0.44	0.39	.680	0.010
Residuals	78	89.44	1.15			
Within-Subjects						
Time Factor	2	3.47	1.73	12.73	<0.001	0.14
IG_CG:Time Factor	2	0.76	0.38	2.77	0.070	0.03
Duration of Diabetes:Time Factor	2	0.20	0.10	0.74	0.468	0.009
Age:Time Factor	2	0.66	0.33	2.43	0.096	0.03
Smoking:Time Factor	4	0.18	0.05	0.33	0.840	0.008
Physical Activity:Time Factor	4	0.12	0.03	0.22	0.917	0.006
Time Factor Residuals	156	21.25	0.14			
Dependent Variables	4	50.63	12.66	12.99	<0.001	0.14
IG_CG: Dependent Variables	4	5.29	1.32	1.36	0.260	0.02
Duration of Diabetes: Dependent Variables	4	2.00	0.50	0.51	0.627	0.007
Age: Dependent Variables	4	2.45	0.61	0.63	0.557	0.008
Smoking: Dependent Variables	8	6.19	0.77	0.79	0.547	0.02
Physical Activity: Dependent Variables	8	2.34	0.29	0.30	0.902	0.008
Dv Factor Residuals	312	304.00	0.97			
Time Factor: Dependent Variables	8	8.14	1.02	7.67	<0.001	0.09
IG_CG:Time Factor: Dependent Variables	8	3.70	0.46	3.49	0.006	0.04
Duration of Diabetes:Time Factor: Dependent Variables	8	0.55	0.07	0.52	0.739	0.007
Age:Time Factor: Dependent Variables	8	2.07	0.26	1.95	0.093	0.02
Smoking:Time Factor: Dependent Variables	16	1.66	0.10	0.78	0.632	0.02
Physical Activity:Time Factor: Dependent Variables	16	1.94	0.12	0.92	0.511	0.02
Time Factor:Dv Factor Residuals	624	82.71	0.13			

SS: type III sum of square; MS: mean of square.

## Data Availability

Not applicable.
